# Evaluating subclinical atherosclerosis, arterial stiffness, and lipid profile in patients with anti neutrophilic cytoplasmic antibodies associated vasculitis: a systematic review and meta-analysis

**DOI:** 10.1097/MS9.0000000000003011

**Published:** 2025-02-07

**Authors:** Rukesh Yadav, Ashish Mishra, Prince Mandal, Susmin Karki, Saurav Pokhrel, Narayan Prasad Neupane

**Affiliations:** Department of Internal Medicine, Maharajgunj Medical Campus, Institute of Medicine, Tribhuvan University, Nepal.

**Keywords:** anti-neutrophilic cytoplasmic antibodies associated vasculitis, arterial stiffness, lipid profile, subclinical atherosclerosis

## Abstract

**Background:**

Cardiovascular diseases (CVD) account for a significant portion of deaths in anti-neutrophilic cytoplasmic antibodies associated vasculitis (AAV). Previous studies have shown the presence of accelerated subclinical atherosclerosis and arterial stiffness in AAV patients that is not solely explained by conventional cardiovascular risk factors. Moreover, lipid profile may be deranged in such patients. The evidence of subclinical atherosclerosis, arterial stiffness, and lipid profile have not been analyzed in AAV patients.

**Methods:**

PubMed, Google Scholar and Embase databases were searched from inception till January 2024 for studies that measured parameters of arterial structure and function ((carotid intimal media thickness (CIMT), flow mediated dilation (FMD), pulse wave velocity (PWV)) and lipid profile of adult patients with AAV and matched healthy controls. Outcomes included standardized mean difference (SMD) with 95% confidence interval (CI) of CIMT, FMD, PWV and lipid profile between AAV patients and healthy controls. The analysis was done via Revman 5.1.

**Results:**

Ten studies comprising 360 AAV patients and 487 healthy controls were included in this analysis. CIMT value was significantly higher in AAV patients as compared to healthy controls [SMD = 0.34, CI = 0.09-0.59, *P* = 0.008, I^2^ = 56%]. Moreover, PWV was significantly higher in AAV patients [SMD = 0.53, CI = 0.26-0.79, *P* < 0.001, I^2^ = 0%] as compared to healthy controls. The lipid profile was not significantly different among the AAV patients and the healthy controls except the total triglycerides level.

**Conclusion:**

This review validates the occurrence of subclinical atherosclerosis and arterial stiffness in AAV patients. Regular assessment using surrogate markers is warranted with aim to reduce CVD in such patients.

## Introduction

Antineutrophil cytoplasmic antibodies (ANCA) associated vasculitis (AAV) include a group of autoimmune diseases, namely granulomatosis with polyangiitis (GPA), microscopic polyangiitis (MPA), and eosinophilic granulomatosis with polyangiitis (EGPA). These disorders are characterized by inflammation and necrosis of small-sized arteries^[1]^.

**HIGHLIGHTS**

- Patients with rheumatological diseases are known to have significant cardiovascular events and often the subclinical atherosclerosis is evident.
- The evidence of subclinical atherosclerosis has been not reviewed adequately in the patients with ANCA associated vasculitis.
- The present meta-analysis reveals significant elevations in CIMT and PWV in patients with ANCA associated vasculitis compared to healthy controls.
- Further large-sampled studies are warranted to evaluate the robustness of our results.

Previous studies have shown the presence of accelerated and subclinical atherosclerosis along with arterial stiffness in AAV patients that cannot be solely explained by conventional cardiovascular risk factors^[2]^. There are studies that highlight increased cardiovascular (CV) events and deaths in AAV patients, attributable to atherosclerosis^[3]^. Endothelial dysfunction, dyslipidemia, and systemic inflammation are three possible explanations for the increased CV events in AAV^[4]^. Moreover, lipid profile may be deranged in such patients. Numerous studies demonstrate the impact of chronic inflammation over time on lipid metabolism^[5]^.

Carotid intima-media thickness (CIMT), flow mediated dilation (FMD), and pulse wave velocity (PWV) are some parameters for assessing arterial structure and function. These parameters have been used, most often CIMT, albeit inconsistently, as surrogate markers for atherosclerosis, even in the initial subclinical stages. Notably, there is a 10%–15% increase in the risk of myocardial infarction and a 13%–18% increase in the risk of stroke for every 0.1 mm increase in absolute CIMT^[6]^. A meta-analysis of 24 studies revealed that CIMT was higher in rheumatic illnesses (rheumatoid arthritis, systemic lupus erythematosus) than in healthy controls^[7]^. The similar meta-analysis regarding CIMT values have not been well analyzed in AAV patients. The CIMT measurement should be used in conjunction with a carotid plaque (CP) evaluation for the best assessment of CV risk in the general population. It is thought that CIMT and CP can anticipate different CV occurrences^[8]^.

FMD measures the dilating capacity of the artery in response to mechanical and chemical stresses. FMD has been linked with endothelial dysfunction and consequent cardiovascular events^[9]^. Patients with long-term inflammatory conditions, such systemic lupus erythematosus, systemic sclerosis, and rheumatoid arthritis, have been shown to exhibit subclinical atherosclerosis^[10,11]^. Meta-analysis regarding the endothelial damage, arterial stiffness and the onset of early atherosclerosis in the context of ANCA vasculitis, such as GPA, EGPA and MPA is currently lacking.

Few research studies have indicated the existence of dyslipidemia in ANCA patients however, the results of these studies have not been consistent^[12]^. The purpose of this study is to evaluate and measure the body of knowledge on arterial stiffness, subclinical atherosclerosis, endothelial damage and lipid profiles in patients with ANCA associated vasculitis. It is necessary for the clinician to detect subclinical atherosclerosis prior to cardiovascular or cerebrovascular events in order to develop preventive treatment for AAV. Thus, we aimed to systematically assess subclinical atherosclerosis, arterial stiffness, and lipid profiles in AAV patients.

## Methods

### Protocol

The Preferred Reporting Items for Systematic Review and Meta-Analysis (PRISMA) criteria were followed in the conduct of this systematic review. The review has been registered in Prospero. Further, the review has been reported in line with AMSTAR (Assessing the methodological quality of systematic reviews) guidelines.

## Search strategy

Systematic literature search was performed to find published studies indexed in PubMed, Google Scholar, and EMBASE databases from their inceptions to January 2024. There was no limitation on the publication date, but filter was applied to search only human studies in English language. No filters for study design or limits for age was considered. The references of selected retrieved articles were also examined. A prespecified search strategy was used to identify the relevant studies. The keywords for the searches were as follows: *carotid intima-media thickness; flow-mediated dilatation, pulse wave velocity, granulomatosis with polyangiitis, Wegener’s Granulomatosis, arterial stiffness, augmentation index, vascular stiffness, aortic stiffness, microscopic polyangiitis, eosinophilic granulomatosis with polyangiitis, Churg Straus syndrome, lipid profile, dyslipidemia, subclinical atherosclerosis, accelerated atherosclerosis, endothelial dysfunction, and ANCA vasculitis.* Variants for all keywords were used to increase the number of studies returned by the search. The references in the identified or related articles were then manually reviewed for other relevant citations. All resulting articles were then screened for CIMT, FMD, PWV, lipid profiles, endothelial dysfunction measures and the inclusion of healthy control groups. If the publications did not contain all the necessary information, missing information was requested directly from the authors. If a study was reported in more than 1 publication, only 1 report was included for the meta-analysis.

## Inclusion criteria

1. Studies having participants with AAV who had arterial stiffness or subclinical atherosclerosis, as measured by pulse wave velocity, intima media thickness or flow-mediated dilatation.
2. All published case-control studies which assessed the arterial stiffness, subclinical atherosclerosis and lipid profile among the AAV patients and healthy controls.

## Exclusion criteria

1. Reviews, case reports, abstracts, non-English studies and animal studies
2. Absence of a healthy control group

## Outcomes measured

Comparison of pulse wave velocity, intima media thickness, flow-mediated dilatation, endothelial dysfunction biomarkers, and lipid profiles between AAV and healthy controls.

## Data extraction

Two authors independently reviewed the titles and abstracts of all the studies that were identified. The inclusion criteria were independently applied to all identified studies, and differences in opinion were resolved by consensus. Full-text versions of potentially relevant papers identified in the initial screening were retrieved, and data concerning study design, the source of information, participant characteristics, AAV type, arterial stiffness assessment, and lipid profile was extracted. In addition, we contacted the authors of the primary reports to request any unpublished data.

## Quality assessment

The methodological quality of the observational studies was evaluated by the two authors using the JBI checklist for case control studies, which is a quality assessment tool for non-randomized studies. The studies were classified as high, moderate and low quality if they scored 8–10, 5–7, and 1–4 in JBI checklist, respectively^[13]^. We did not exclude studies of poor quality based on this scoring system. Discrepant opinions between the authors were resolved by consensus.

## Data synthesis

We calculated the pooled standardized mean difference (SMD) of the PWV, CIMT, FMD of arterial stiffness, endothelial dysfunction parameters, and lipid profiles between the AAV patients and the healthy controls. All outcomes were reported as effect estimates and 95% confidence intervals (CIs), comparing AAV and normal groups by random or fixed effects modeling. We presented the results narratively (qualitative analysis) if there was insufficient data available for calculating pooled effect size. The heterogeneity of effect estimates across these studies was quantified using the Q statistic and I^2^ (*P* <0.05 being considered statistically significant). The I^2^ statistic is the ratio of the true heterogeneity to the total observed variation, and an I^2^ value of 30% to 60% indicates the presence of moderate heterogeneity, 50% to 90% indicates substantial heterogeneity, and 75% to 100% indicates considerable heterogeneity. Publication bias was assessed using a funnel plot. Forest plot was used to depict the results of the meta-analysis. Meta-analysis was performed by using Review Manager Version 5.4. Subgroup analysis was performed for GPA, EGPA, and MPA patients independently whenever possible. Moreover, sensitivity analysis was done when the analyses revealed considerable heterogeneity.

## Results

### Studies and search results

**Figure 1.**
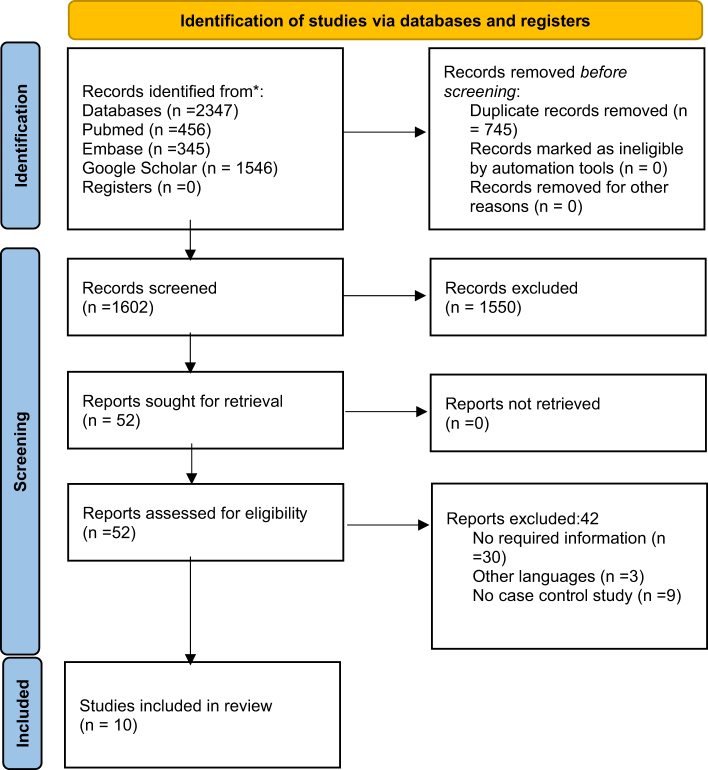
PRISMA flow diagram showing the detailed strategy for the study selection.

2347 studies were obtained from the search databases for screening, and duplicate entries in the bibliographic databases were eliminated. Two authors assessed the remaining studies for title and abstract. Then, each of the authors conducted a full-text review of a chosen set of studies based on inclusion and exclusion criteria. Ten studies in all were selected for review. Discussion and consultation were done to settle any disagreements. The strategy for studies selection has been outlined in Figure 1.

**Figure 2.**
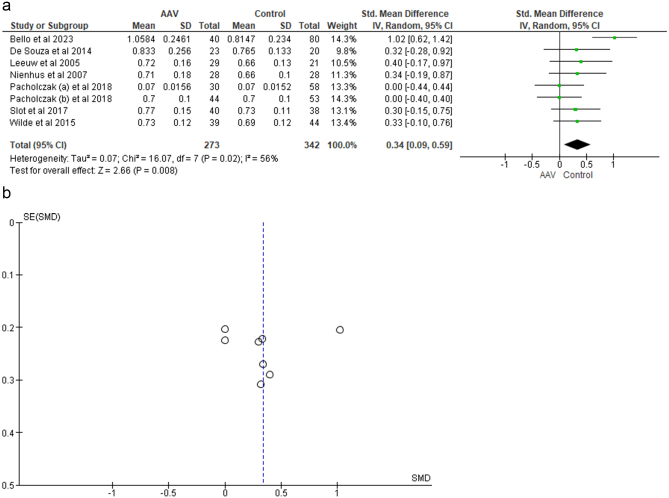
(a) Forest plot showing the comparison of CIMT among AAV patients and healthy controls. (b) Funnel plot for publication bias detection (CIMT analysis).

### Study characteristics

We included ten case control studies among which five were conducted in the Netherlands^[14-18]^, two in Poland^[12,19]^ and the remaining in Italy, UK and Turkey^[20-22]^. A total of 360 AAV cases and 487 controls were included in this review. 54.4 % of the cases and 52.5 % of the controls were male. The age of the included population ranged from 40 to 69 years. There was a total of 227 GPA cases, 95 EGPA cases, 27 MPA cases, and 11 AAV unspecified cases. The detailed study characteristics is outlined in Table 1.

**Table 1:** Baseline characteristics of the included studies

Author	Country	Study design	AAV type	Sample size (N)		Age (Mean ± SD/Range)		Sex (Male/Female)		Disease Duration (Months) (Mean ± SD/Range)	Treatment Drugs (N)
				Case	Control	Case	Control	Case	Control		
Leeuw et al, 2004	Netherland	Case-control	GPA	29	26	53.2	53.2	19/10	16/10	59 (26–124)	HMG-CoA inhibitor Antihypertensive^[24]^ Prednisolone^[25]^
Nienhuis et al, 2007	Netherland	Case-control	GPA	28	28	50 ± 9	49 ± 9	17/11	17/11	91 (60–124)	Anti-hypertensive ^[18]^, HMG-CoA inhibitor^[2]^
De Souza et al, 2014	Netherland	Case-control	GPA	23	20	55.2 (45.7–62.4)	49.8(43.0–57.4)	14/9	11/9	131.2 ± 60.2	Atorvastatin and Prednisolone
Pacholczak et al(a), 2018	Poland	Case-control	GPA	44	53	59(46–61)	48(43–61)	21/23	22/23	54(12–108)	Steroid^[26]^, Azathioprine:12, Cyclophosphamide: 37 Methotrexate: 5 Mycophenolate mofetil: 2 Rituximab: 13
Slot et al, 2017	Netherland	Case-control	GPA, EGPA, MPA	40	38	56 ± 10.5	54.4 ± 9.7	22/18	17/21	31.2 (7.2–195.6)	Prednisolone, Cyclophosphamide, Azathioprine
Yildiz et al, 2007	Turkey	Case-control	GPA	5	5	45 ± 15.1	44 ± 14.7	4/1	4/1	3.4 ± 2.7(years)	Prednisolone, Cyclophosphamide, Azathioprine
Farrah et al, 2022	UK	Case-control	GPA, EGPA, MPA	32	32	55 ± 13	54 ± 12	23/9	23/9	NA	Aspirin, ACE I or ARB, CCB, Beta blocker, Alpha blocker, Diuretic, Statin, Ezetimibe
Bello et al, 2023	Italy	Case-control	EGPA	40	80	59(48–71)	56(45–69)	16/24	33/47	NA	Corticosteroid, Intravenous immunoglobulins, Rituximab, Azathioprine, Cyclosporin, Mycophenolate, Methotrexate, Omalizumab
Pacholczak et al(b), 2018	Poland	Case-control	EGPA	30	58	49(44–58)	47.5(40.5–58.5)	10/20	24/34	54(36–96)	Corticosteroid^[23]^ ACEI/ARB^[11]^ Statins^[6]^ Beta-blockers^[8]^ Diuretics^[8]^ Calcium channel blockers^[4]^
Wilde et al, 2015	Netherland	Case-control	GPA, EGPA,MPA	39	44	56 ± 11	51 ± 13	21/18	18/26	103 ± 79	Azathioprine, Methotrexate, Mycophenolate mofetil/Mycophenolic acid, Steroid, Cyclophosphamide

AAV, anti neutrophilic cytoplasmic antibodies (ANCA) associated vasculitis; GPA: granulomatosis with polyangiitis; EGPA, eosinophilic granulomatosis with polyangiitis; MPA, microscopic polyangiitis; HMG-CoA, hydroxy-methyl-glutaryl-coenzyme A; ACEI, angiotensin-converting enzyme inhibitors; ARB, angiotensin receptor blockers; NA: not available.

## Quality assessment results

Eight studies were of high quality and only two studies were judged to have moderate quality. The quality assessment result is summarized in Table 2.

**Table 2:** Quality assessment result using JBI checklist

Authors	Comparable of the group	Appropriate of matching case and control groups	Same criteria used for identification of cases and controls	Exposure measured in a valid and reliable way “gold standard”	Exposure measured in the same way for cases and controls	Confounding factors	Dealing with confounding factors	Outcomes measured in a valid and reliable way	Exposure period of interest long enough to be meaningful	Appropriate statistical analysis	Scores (Out of 10)	Quality (low, moderate, high)
Leeuw et al, 2004	Yes	Yes	Yes	Yes	Yes	No	No	Yes	Yes	Yes	8	High
Nienhus et al, 2007	Yes	Yes	Yes	Yes	Yes	No	No	Yes	Yes	Yes	8	High
De Souza et al, 2014	Yes	Yes	Yes	No	No	No	No	Yes	Yes	Yes	6	Moderate
Pacholczak (a) et al, 2018	Yes	Yes	Yes	Yes	Yes	Yes	Yes	Yes	Yes	Yes	10	High
Yildiz et al, 2007	Yes	No	Yes	Yes	Yes	Yes	No	No	Yes	Yes	7	Moderate
Slot et al, 2017	Yes	Yes	Yes	Yes	Yes	Yes	No	Yes	Yes	Yes	9	High
Farrah et al, 2022	Yes	Yes	Yes	Yes	Yes	Yes	No	Yes	Yes	No	8	High
Pacholczak (b) et al, 2018	Yes	Yes	Yes	Yes	Yes	Yes	Yes	Yes	Yes	Yes	10	High
Bello et al, 2023	Yes	Yes	Yes	Yes	Yes	Yes	No	Yes	Yes	Yes	9	High
Wilde et al, 2015	Yes	Yes	Yes	Yes	Yes	No	No	Yes	Yes	Yes	8	High

Low: 1–4, moderate: 5-7, high: 8–10.

## Outcomes of interest

### CIMT

Eight studies provided data on CIMT. In all these studies, the measurements were done using carotid ultrasonography. While most of the studies included measurements from both the carotid arteries and/or their branches, two studies, those by *Leeuw et al*^[14]^ and *Nienhus et al*^[15]^, involved measurement of intima media thickness (IMT) on only the left common carotid artery (CCA). Even among the studies which included measurements of both sides, there were some discrepancies in the exact site and number of measurements. *Pacholczak et al*^[12]^ measured the IMT on anterior and posterior walls of both the CCAs, while *Bello et al*^[22]^ also included measurement of a third lateral wall. *De souza et al*^[18]^included measurements from the carotid bulbus, CCA and internal carotid artery (ICA). *Slot et al*^[16]^ and *Wilde et al*^[17]^ took measurements of the IMT of the far walls of the CCA viewed on 4 different angles using Meizer’s Arc over a predefined 10 mm segment. The studies including measurements on only one side, that is those by *Leeuw et al*^[14]^ and *Nienhus et al*^[15]^, took measurements of the left common carotid artery only, at three different positions 1 cm proximal to the bulbus. In all the eight studies, mean IMT was calculated from the multiple measurements taken at different sites. *Leeuw et al*^[14]^ also included measurements using wall track system (WTS). CIMT was significantly different between AAV patients and healthy controls [SMD = 0.34, CI = 0.09-0.59, *P* = 0.008, I^2^ = 56%] as shown in Figure 2a. After removing the study by *Bello et al*, the heterogeneity reduced to 0%, but the analysis remained statistically significant [SMD = 0.21, CI = 0.03-0.39, *P* = 0.02, I^2^ = 0%]^[22]^. Upon subgroup analysis for GPA patients, no significant differences in CIMT between cases and controls were found [SMD = 0.21, CI = −0.09 to 0.46, *P* = 0.10, I^2^ = 0%]. For EGPA patients, subgroup analysis revealed no significant difference in CIMT value as compared to healthy controls [SMD = 0.34, CI = −0.33 to 1.02, *P* = 0.32, I^2^ = 87%]. These non-significant results could be due to the smaller number of the studies and the patients of the GPA and EGPA subgroups. Funnel plot of the analysis revealed publication bias as in Figure 2b.

### PWV

**Figure 3.**
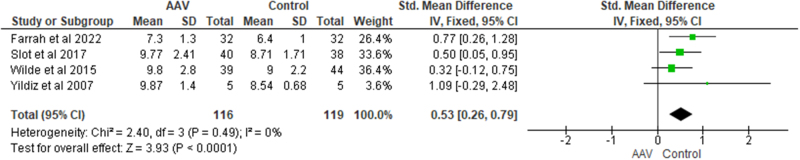
Forest plot showing the comparison of PWV among AAV patients and healthy controls.

Data for PWV was available from four studies. In the study by *Yildiz et al*^[20]^, pulse pressure waveforms were recorded in the right common carotid and femoral arteries. The pulse wave velocity was calculated by the distance travelled by the pulse, given by the distance between the two recording sites measured on the surface of the body and the time delay between the pulse waveforms in the two recordings. Studies by *Wilde et al*^[17]^ and *Slot et al*^[16]^ also included pulse wave velocity between carotid and radial artery (representing the brachial tract) in addition to the carotid-femoral pulse wave velocity (representing the aortic tract). *Slot et al* also corrected the PWV values for MAP. *Farrah et al*^[21]^. have not included the method of measurement of PWV in their study. AAV patients had significantly higher PWV as compared to healthy controls [SMD = 0.53, CI = 0.26-0.79, *P* < 0.001, I^2^ = 0%] (Fig. 3). The analysis revealed no heterogeneity.

**FMD**: In the study by *Pacholczak (a) et al*, EGPA patients had a 38.8% lower relative increase of FMD (FMD%) (*P* < 0.001) as compared to healthy controls indicating endothelial dysfunction^[12]^. Even after accounting for possible confounders, GPA patients’ FMD% decreased by 48.9% as compared to controls (*P* < 0.001) in the study by *Pacholczak (b) et al*^[19]^.

**Aortic index (AI)**: In the study by *Farrah et al*, the patients with AAV had increased AI as compared to the healthy controls (26% ± 11% vs. 20% ± 10%, *P* = 0.031)^[21]^.

## Carotid atherosclerosis and carotid plaques

Carotid plaques were defined to be present when IMT was 1.3 mm or more.

*De Souza et al* found no difference in the prevalence of carotid plaques among GPA patients and the healthy controls^[18]^. However, the study by *Bello et al* in EGPA patients, the prevalence of carotid plaques was significantly higher in EGPA patients (42.5%) as compared to 13.75% in healthy controls^[22]^.

## Lipid profile and body mass index (BMI)

**Figure 4.**
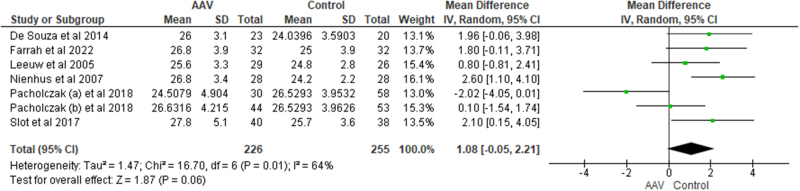
Forest plot showing the comparison of BMI among AAV patients and healthy controls.

**BMI**: Data for BMI was available in seven studies. BMI was higher in the AAV patients however, the statistical significance was not achieved [MD = 1.08, CI = −0.05 to 2.21, I^2^ = 64%, *P* = 0.06] (Fig. 4). The difference became significant after excluding *Pacholczak (b) et al* and heterogeneity dropped to 20% [MD = 1.53, CI = 0.82 to 2.24, I^2^ = 20%, *P* < 0.0001]^[19]^. After subgroup analysis, BMI was significantly higher in GPA patients as compared to healthy controls [MD = 1.37, CI = 0.54 to 2.20, I^2^ = 47%, *P* = 0.001].

**Figure 5.**
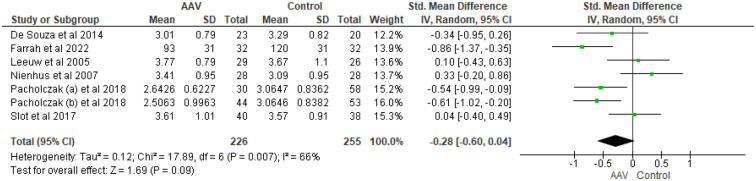
Forest plot showing the comparison of LDL among AAV patients and healthy controls.

**Low-density lipoprotein cholesterol** (**LDL-C**): LDL-C values for analysis were available in seven studies. LDL-C level was not significantly different among AAV patients and the healthy controls. [SMD = −0.28, CI = −0.60 to 0.04, I^2^ = 66%, *P* = 0.09] (Fig. 5). The difference remained non-significant for GPA patients after subgroup analysis [SMD = −0.14, CI = −0.59 to 0.31, I^2^ = 67%, *P* = 0.53].

**Figure 6.**
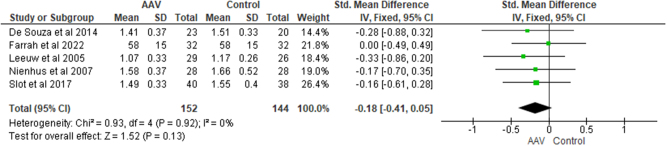
Forest plot showing the comparison of HDL among AAV patients and healthy controls.

**High-density lipoprotein cholesterol** (**HDL-C**): Meta analysis that included 296 people from five studies revealed no significant difference in HDL-C level between AAV patients and healthy controls [SMD = −0.18, CI = −0.41 to 0.05, I^2^ = 0%, *P* = 0.13] (Fig. 6). Similarly, GPA patients did not have significantly different HDL-C levels [SMD = −0.26, CI = −0.58 to 0.06, I^2^ = 0%, *P* = 0.11].

**Figure 7.**
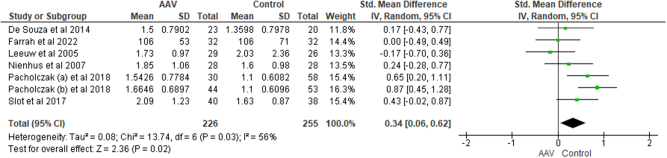
Forest plot showing the comparison of TG among AAV patients and healthy controls.

**Triglycerides (TG**): Meta-analysis of 226 AAV patients and 255 healthy controls revealed significantly higher TG levels in AAV patients as compared to healthy controls [SMD = 0.34, CI = 0.06 to 0.62, I^2^ = 56%, *P* = 0.02] (Fig. 7). However, after subgroup analysis the TG levels were not significantly different between GPA patients and healthy controls [SMD = 0.30, CI = −0.17 to 0.77, I^2^ = 70%, *P* = 0.21].

**Figure 8.**
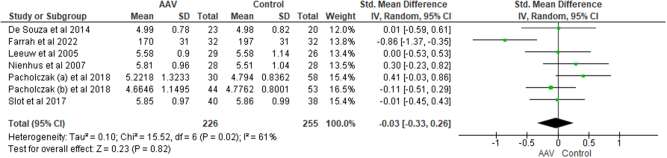
Forest plot showing the comparison of TC among AAV patients and healthy controls.

**Total cholesterol (TC**): Meta-analysis of seven studies revealed no significant difference in total cholesterol level among AAV patients and the healthy controls [SMD = −0.03, CI = −0.33 to 0.26, I^2^ = 61%, *P* = 0.82] (Fig. 8). Subgroup analysis revealed similar non-significant results between GPA patients and the healthy controls [SMD = 0.02, CI = −0.22 to 0.27, I^2^ = 0%, *P* = 0.85].

### Vascular endothelial markers

**Figure 9.**
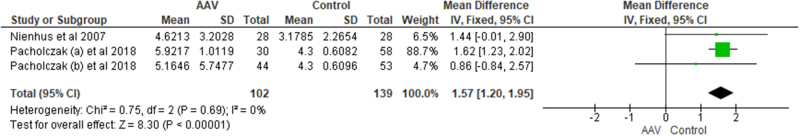
Forest plot showing the comparison of TM among AAV patients and healthy controls.

Three studies provided data for VCAM and thrombomodulin (TM) level for the meta-analysis. The analysis revealed significantly higher TM levels in the AAV patients as compared to healthy controls [MD = 1.57, CI = 1.20 to 1.95, I^2^ = 0%, *P* < 0.00001] (Fig. 9). The difference remained significant for GPA patients as well [MD = 1.20, CI = 0.09 to 2.31, I^2^ = 0%, *P* = 0.03].

**Figure 10.**
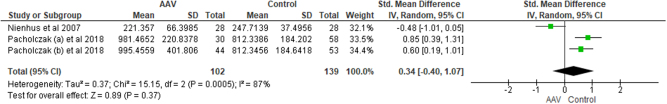
Forest plot showing the comparison of VCAM among AAV patients and healthy controls.

However, the VCAM level was not significantly different between AAV patients and the healthy controls [SMD = 0.34, CI = −0.40 to 1.07, I^2^ = 87%, *P* = 37] (Fig. 10).

### Discussion

The present meta-analysis reveals significant elevations in CIMT and PWV in patients with ANCA associated vasculitis as compared to healthy controls. These findings align with similar observations in other chronic inflammatory rheumatic diseases^[28]^. CIMT and PWV are established markers for assessing subclinical atherosclerosis and arterial stiffness, respectively^[29,30]^. Arterial stiffness and atherosclerosis are predictive of cardiovascular risk in rheumatic diseases^[23,28]^, including AAV where a notably high frequency of cardiovascular events has been reported^[25]^.Consequently, the most recent guideline from EULAR regarding AAV advises conducting periodic assessments to evaluate cardiovascular risk in patients diagnosed with AAV^[31]^.

CIMT measurements from the posterior wall of CCA have been shown to better predict future cardiovascular events^[24]^. Measurement of CIMT in the anterior wall is more challenging and difficult to reproduce and thus, does not reliably predict the future cardiovascular events. Hence, guidelines for measuring CIMT, like the Mannheim consensus and the American Society of Echocardiography’s consensus, recommend measurement on the posterior wall^[24]^. While many guidelines recommend the use of IMT at CCA only for the risk prediction for CVD, it has been seen that IMT measurement at all three segments (carotid bulbus, CCA, and ICA) enhances the risk prediction^[32]^. There was heterogeneity in terms of CIMT measurements in these regard among the included studies in this meta-analysis.

In this meta-analysis, the difference in CIMT between AAV patients and control was found to be 0.05 mm. This is clinically relevant as even 0.01 mm increase in CIMT can substantially increase the risk of myocardial infarction and stroke^[6]^. Subgroup analysis of GPA and EGPA patients did not exhibit significant differences in CIMT compared to healthy controls. The results were contrasting among the included studies. This could be because of the limited studies reporting CIMT in these subgroups, smaller sample size, and inadequate duration of follow up of the patients in these studies. Further, the CIMT value is known to have negative correlation with cumulative dose and duration of corticosteroids use. Aggressive immunosuppressive therapy and corticosteroids, given to prevent the relapse of the disease, might have influenced the CIMT value as well^[15]^.

Although CIMT value is an established marker of subclinical atherosclerosis, its progression over time has not been validated to be the marker for atherosclerosis progression. There are some studies which show correlation between the two, while others show that correlation is not significant. A study by *Bello et al* found that the common carotid intimal media thickness value correlated with disease duration in patient with EGPA^[22]^. Similar relation between GPA time course and CIMT value was observed by *Pacholczak (b) et al* 2018^[19]^. However, *K de Leeuw et al* did not find significant difference in CIMT progression rate among AAV and healthy controls. This unexpected result could be due to inclusion of AAV patients with no active disease and aggressive treatment of traditional risk factors due to the increased awareness of atherosclerosis^[33]^.

For subgroups like GPA and EGPA patients who did not exhibit significant differences in CIMT as compared to healthy controls, FMD could be a potential marker. A study by *Pacholczak* (a) *et al* with EGPA patients demonstrated a substantial decrease in flow-mediated dilation (FMD%), indicative of endothelial dysfunction^[12]^. This could suggest a distinct pattern of cardiovascular involvement in this subgroup. Systemic inflammation in vasculitis predisposes to endothelial dysfunction^[34]^. This is supported by resolution of endothelial dysfunction following administration of infliximab to AAV patients^[33]^. In AAV, there is activation of the primed neutrophils, macrophages, and the complement system, along with the release of inflammatory cytokines and reactive oxygen species^[35,36]^. This causes an endothelial dysfunction which is characterized by reduced nitric oxide and increased endothelin-1 production. This ultimately leads to arterial stiffness and possibly decreased FMD^[36]^.

On analysis of endothelial dysfunction, TM level was significantly higher in AAV patients as compared to healthy controls. However, VCAM-1 level was not significantly different according to the meta-analysis. *K. de Leeuw et al* found the positive correlation between CIMT value and VCAM-1 signifying the relation between the endothelial activation and atherosclerosis^[14]^.

On analysis of the lipid profile, no significant difference in LDL-C and total cholesterol between the AAV patients and healthy control was observed in this meta-analysis. However, the studies done in other inflammatory conditions suggest that LDL-C and total cholesterol remains on the lower side^[26,27]^. Post hoc analysis of the rituximab for AAV by *Wallace et al* identified that lipid profile decreases in the active disease state with active inflammation^[37]^. Contrary to the general population, this low lipid level correlates with a higher cardiovascular risk, a phenomenon known as the “lipid paradox”^[38]^. Although the exact mechanisms of lipid paradox in inflammatory conditions are not fully understood, proinflammatory cytokines such as IL-6 and TNF-α have been shown to increase the expression of LDL and SR-B1 receptors. This, in turn, enhances the liver’s uptake of LDL and promotes the secretion of cholesterol into the bile, resulting in lower circulating LDL levels^[39]^. The lipid profile analysis also indicated elevated TG levels in AAV patients compared to healthy controls. This observation is consistent with findings in RA patients^[40]^. This suggests that dyslipidemia, particularly elevated TG levels, may be associated with AAV and contributes to the development of accelerated atherosclerosis by modulating inflammation, oxidative stress and foam cell formation^[41]^. Although not significant, LDL-C levels were found to be lower in AAV patients as compared to controls. This could be due to the fact that the AAV patients were more frequently treated with lipid lowering therapies to prevent possible cardiovascular risk. In the studies used for this analysis, 2-60% of patients were under lipid lowering therapies due to increased cardiovascular risk. Statin treatment is considered as standard unless it is contraindicated^[34]^.

This is the first meta-analysis to comprehensively analyze subclinical atherosclerosis, arterial stiffness, and lipid profile in AAV patients. Although the meta-analysis revealed a significant difference in CIMT values between AAV patients and healthy controls, individual studies showed non-significant or marginally significant differences This could be due to different factors like level of chronic inflammation in each AAV subtype, methods of CIMT measurements, duration of the AAV, active or inactive stage of the disease, effect of corticosteroids or other immunosuppressants, and other patient’s demographic factors like traditional cardiovascular risk factors.

## Limitations and future implications

This study has some limitations. First, the sample size of the included patients was considerably low. Some of the analyses revealed considerable heterogeneity. Thus, results of the analysis should be cautiously interpretated. The heterogeneity in the findings can be attributed to methodological differences and variations in population selection criteria across the studies. For example, in the measurement of CIMT, while most of the studies report measurements from both the carotid arteries and/or their branches, the studies by *Leeuw et al*^[14]^. and *Nienhus et al*^[15]^ report measurement of IMT on only the left CCA. Even among the studies which included measurements of both sides, there were some discrepancies in the exact site, and number of measurements. Similarly, in assessing PWV *Yildiz et al*^[20]^ recorded pulse pressure waveforms in the right common carotid and femoral arteries whereas studies by *Wilde et al*^[17]^. and *Slot et al*^[16]^. also included pulse wave velocity between carotid and radial artery (representing the brachial tract) in addition to the carotid-femoral pulse wave velocity (representing the aortic tract). Furthermore, variations in participant demographics (age, gender, ethnicity) as well as differences in the intervention’s type, duration, intensity, or delivery method and follow-up may have contributed for inconsistent results. Given these limitations, future studies should focus on incorporating larger sample sizes to enhance statistical power and reduce heterogeneity. Longitudinal data would be valuable in elucidating the relationship between subclinical atherosclerosis and cardiovascular events in AAV patients. Additionally, the inclusion of more specific biomarkers could improve the understanding of these relationships. Future research should also consider randomized controlled trials (RCTs) to assess the impact of targeted therapies on cardiovascular outcomes in AAV patients, as this could provide more definitive evidence regarding effective management strategies for reducing atherosclerosis and cardiovascular risk in this population.

## Conclusion

This review validates the occurrence of subclinical atherosclerosis in AAV patients. Regular assessment using surrogate markers is warranted. Further, special care is required to be taken to reduce the traditional cardiovascular risk factors in AAV patients.

## Data Availability

Not applicable.

## Research registration unique identifying number (UIN)

PROSPERO Registration ID: CRD42024507156.
